# Examining Potential Implicit Bias in Oncologist-Patient Communication (CONNECT): Protocol for an Observational 2-Site Study

**DOI:** 10.2196/66086

**Published:** 2025-08-14

**Authors:** Veronica C K Duck, Marsha L Augustin, Jose A Morillo, Aviel N Alkon, Robert M Thomas, Brianna N Richardson, Lihua Li, Kathryn I Pollak, Cardinale B Smith

**Affiliations:** 1 Department of Population Health Sciences Duke University School of Medicine Duke Cancer Institute Durham, NC United States; 2 Division of Hematology and Medical Oncology Tisch Cancer Institute, Icahn School of Medicine at Mount Sinai Icahn School of Medicine at Mount Sinai New York, NY United States; 3 Division of General Internal Medicine Duke University School of Medicine Durham, NC United States; 4 Cancer Prevention and Control Duke Cancer Institute, Duke University Duke Cancer Institute Durham, NC United States

**Keywords:** oncology, communication, oncologist-patient communication, audio-recorded encounters

## Abstract

**Background:**

Compared with White patients, minoritized patients (Black and Hispanic patients) have a higher incidence of advanced solid cancers and have a higher mortality. These patients also report poor patient-centered communication and worse pain assessment and management. Although many factors contribute to these disparities, physician implicit bias may be a contributing factor.

**Objective:**

The primary goal of this study is to evaluate the role of implicit bias among oncologists and examine the impact on racial or ethnic differences in objective assessments of communication with minority patients with advanced cancer.

**Methods:**

To accomplish this goal, we plan to recruit 65 oncologists and 325 patients (5 patients per oncologist) with advanced solid cancer from ambulatory cancer clinics within the diverse settings of the Mount Sinai Health System in New York City and the Duke University Health System in Durham, NC. We audio record patient-oncologist encounters during a postimaging visit, with 3 encounters for each of the patients. We will analyze the recorded visits and compare the patient-centered communication content of these conversations. Immediately after the recorded visit (no more than 2 weeks later, in order to minimize recall bias), patients are required to complete a follow-up survey to evaluate patient-centered outcomes. A 3-month follow-up survey is used to assess pain levels and control, use of analgesics, and psychological distress. A 6-month follow-up survey is used to assess psychological distress. We administer the Implicit Association Test to oncologists to assess their level of implicit bias toward patients who identify as Black or Hispanic after we finish recording patient encounters.

**Results:**

Funding from the National Cancer Institute was received in March 2021. Patient and oncologist recruitment began in March 2022. We have recruited all 65 oncologists in the study, and patient recruitment is ongoing. The study team plans to continue to enroll patients until March 2025. As of December 2024, we have enrolled 245 patients. We expect to publish the findings in October 2026.

**Conclusions:**

In this paper, we outline the study methods, describe the development of a codebook to assess pain conversations being used to evaluate primary and secondary outcomes, and discuss challenges and lessons learned throughout the study.

**International Registered Report Identifier (IRRID):**

DERR1-10.2196/66086

## Introduction

Compared with White patients, minoritized patients (Black and Hispanic patients) have a higher incidence of advanced solid cancer and have a higher mortality rate [1,2]. Although many factors contribute to such disparities, physician implicit bias may be a contributing factor [3]. Moreover, disparities have been reported in the outcomes of minoritized patients with advanced solid cancer [4,5]. Rather than improving over time, these inequities have been widening [6]. Multiple studies have shown that minority patients with advanced cancer have inadequate discussions about treatment, prognosis, and goals of care [7,8] and are more likely to have suboptimal pain assessment and management [9]. These disparities translate into substandard cancer treatment, worse quality of life, and poorer survival among these patients than among White patients [4,5,9-12]. Thus, there is a critical need to address these gaps for patients with advanced cancer. Although patient- and system-level factors contribute to cancer disparities, physician-related factors, such as limited cultural competence and bias, also likely play relevant roles. Unlike the rare case of explicit bias, implicit bias occurs at an unconscious, unintentional level, where people make judgments about others based on their groups, rather than considering them as individuals. Although many researchers have discovered race disparities in communication [13], few have examined the role of implicit bias, a potentially modifiable factor. As such, 2 Institute of Medicine reports, *Unequal Treatment* and *Relieving Pain in America*, strongly recommend evaluation of the role of implicit bias in disparities [3,14]. However, almost no researchers have examined bias in oncology encounters.

The impact of implicit bias on the outcomes of ethnic minority patients with advanced cancer may be associated with different mechanisms. Minority patients have reported poor patient-centered communication (eg, respecting and responding to patients’ wants, needs, and preferences), which can influence assessments, recommendations, and outcomes [15]. Implicit bias could be an underlying barrier to patient-centered communication, thus increasing the chance that ethnic minority patients receive care that is not standard or discordant with preferences and values, which can lead to less satisfaction with care [16-18] and increased psychological distress [19]. Additionally, there are disparities in pain assessment and management, which may also be related to the implicit bias of physicians [3]. Most previous studies specifically examined bias toward Black people as compared to White people [20], and only 4 studies included Hispanic people [20]. Further, most studies had a small number of participants and were focused on trainees (ie, medical students or residents) and not physicians in practice [21,22]. Prior research has focused on Black, noncancer populations and primarily used hypothetical patient vignettes [17,18]. However, significant knowledge gaps exist regarding the role of implicit bias in actual communication. Building on our prior research [23], we propose to confirm the extent of implicit bias among oncologists and examine how it affects objective and subjective outcomes, such as discussions of pain, pain control, and pain management, along with patient satisfaction with communication and psychological distress among ethnic minority patients with advanced cancer. This study will collect one of the largest datasets of conversations between oncologists and Black and Hispanic patients to date and will serve as the foundation for an intervention for oncologists and cancer patients. The specific aims of this study are to (1) evaluate the association of oncologists’ implicit bias with racial or ethnic differences in objective assessments of patient-centered communication in oncologist-patient encounters; (2) examine the association of oncologists’ implicit bias in explaining racial or ethnic differences in the subjective outcomes of psychological distress and satisfaction with communication among patients with advanced solid cancer; and (3) assess if implicit bias explains racial or ethnic disparities in oncologists’ assessment of pain, use of guideline-concordant pain management, and pain control among patients with advanced solid cancer.

## Methods

### Ethics Approval

This study has been reviewed by the Icahn School of Medicine Protection of Human Subjects/Institutional Review Board (PPHS/IRB) and the Duke University Health System (DUHS) IRB, and it has been determined that the study procedures are in accordance with ethical standards for experiments on human subjects (Study-21-00396/IRB Pro00108633). We obtain informed written consent using an IRB-approved informed consent document. Subjects are free to withdraw from participation in the study at any time upon request. To preserve subject confidentiality, subjects are assigned coded study ID numbers. Using these study ID numbers, none of the collection forms will contain the names or medical record numbers of the subjects or other personal identifiers. We pay participants for their time and effort (enrollment [visit 1]: US $30, visit 2: US $15, and visit 3: US $15).

### Overview

This study is a prospective 2-site observational trial that examines postscan encounters between oncologists and their patients with advanced solid cancer who have a prognosis of less than 2 years. We plan to enroll 65 oncologists and approximately 325 patients (4-7 patients per oncologist; with relatively equal proportions of those who identify as White, Black, and Hispanic) seen at outpatient cancer clinics at the Mount Sinai Health System (MSHS) and DUHS. We collect up to 21 audio recordings of encounters per oncologist, with 3 encounters for each of the 4-7 patients, and code the audio-recorded encounters.

### Oncologist Recruitment

Study recruitment has occurred in waves over the past 3 years. We have introduced oncologists to the study and approached them during clinician meetings or individually. Oncologists are eligible if they provide care to patients with advanced solid cancer at MSHS or DUHS for at least a half-day per week and will continue practicing at either facility for at least 1 year.

### Patient Recruitment

Before patient recruitment, the study staff had visited clinics to introduce the study to the clinic staff. This study includes patients with advanced solid cancer who are 21 years or older; are on active treatment (ie, chemotherapy, targeted therapy, and immunotherapy); identify as Black/African American, White, or Hispanic; are English-speaking; and are scheduled for a postscan visit. Patients are excluded if they are diagnosed with more than one malignancy (except for nonmelanoma skin cancer) in the last 5 years. Patients are identified via the electronic health record (EHR; both sites use Epic) from the schedules of consented oncologists and are sent a letter from their oncologist describing the study, with an opt-out number for declining to participate in the study. We attempt to recruit equal numbers of White, Black, and Hispanic patients.

### Data Collection

#### Oncologists

We obtain oncologists’ consent either through paper consent in the clinic by the study staff or via email through REDCap. We collect oncologist baseline surveys (see Multimedia Appendix 1) prior to the enrollment of the first patient via REDCap. We also collect a postencounter survey (see Multimedia Appendix 2) from oncologists after 1 of the 3 audio-recorded encounters. These surveys assess demographics, practice characteristics and patterns, and opioid prescription practices. After recording all encounters for the oncologists, we assess implicit bias using the Implicit Association Test (IAT) [19], which evaluates the level of implicit bias toward those who identify as Black or Hispanic. The IAT measures the strength of the association between concepts and stereotypes or evaluations [19]. We send oncologists a REDCap link with their IAT test. In a recent meta-analysis, 14 of 15 studies identified the existence of implicit bias against minorities among health professionals [24].

#### Patients

After sending recruitment letters, we call, email, or text potentially eligible patients to screen them. After the staff determine that patients are eligible (see Multimedia Appendix 3), we obtain consent either electronically via email through REDCap or in person during their encounter. We ask them to complete a baseline survey (see Multimedia Appendix 4) before their appointment and audio record their encounter. We use an innovative process of collecting all data on an iPad and implement a temporary lock on the iPad to prevent stopping or editing of the recording. Patients are required to complete a postaudio survey (see Multimedia Appendix 5) immediately after their appointment to assess pain levels and control, use of analgesics, ease of access to opioids, use of navigation or care coordination services, psychological distress, and satisfaction with communication. We store all consent forms, surveys, and audio recordings in REDCap. We clean and transcribe the audio recordings.

### Outcomes

Our primary outcome is patient-centered communication, which we assess from coding audio-recorded encounters, open questions, reflective statements, empathic responses to empathic opportunities, and eliciting questions. Our secondary outcome involves discussions of pain, use of guideline-concordant pain management, and pain control, as well as satisfaction and psychological distress among patients with advanced solid cancer. We assess this through coding discussions of pain and goals of care conversations in audio-recorded encounters and postencounter surveys completed by patients after their postscan visit.

### Data Analysis

To assess if implicit bias causes, in part, racial disparities in patient-centered communication, we use multilevel structural equation modeling, which allows for simultaneously examining several regression-type relationships while accounting for latent variables. Specifically, we assess models of complete versus partial mediation by comparing constrained models (direct effects constrained to zero) with unconstrained models that allow for partial mediation [25].

We use 3 established codebooks and 1 newly developed codebook with definitions and examples for each code. These codes include measures of patient-centered communication, goals of care, and pain. Four teams of 2 coders each code all transcripts for our primary and secondary outcomes. Coders listen to the audio-recorded encounters between the oncologists and their consented patients to record instances of goals of care conversations, discussions of patient-perceived pain, discriminatory behaviors, patient participatory behaviors, and effective communication skills (eg, open questions and empathic statements). All 8 coders are trained by the investigator through reviewing audio recordings until interrater reliability is achieved. Interrater reliability is achieved by ensuring that each coder is coding with at least 80% similarity to the other coder on their team. All teams are monitored regularly by the investigators.

The first codebook includes aspects related to our primary outcome (open questions, reflective statements, empathic responses to empathic opportunities, and eliciting questions) [26] and the REMAP (reframe, empathic opportunity, map out patient values, align with values, and propose a plan) framework for the goals of care conversations (eg, aligning with patient values and prognosis) [27]. The second codebook includes 6 global ratings for oncologist communication (ie, flow, attentiveness, warmth, respect, concerns, and rushed) and 1 global rating for patient communication (eg, guarded open). The third codebook includes codes for patient-perceived discriminatory behaviors (eg, stereotyping) [28] and patient participatory behaviors (eg, question asking and assertive responses) [29]. The fourth codebook includes codes assessing discussions of pain between the patient and oncologist (eg, initiates pain discussions, perceived impact of pain, and associated symptoms). Prior to the creation of the fourth codebook, the team used 2 frameworks (the SHARE [seek, help, assess, reach, and evaluate] approach [30] and the SOCRATES tool for clinical pain assessment [31]) to conduct a qualitative data analysis by coding 30 transcripts for discussions of pain, which allowed them to establish codes for the codebook based on common elements.

### Statistical Power and Sample Size

To detect the effect size (relative risk of 0.80; proposed 20% difference) and assume an equal size of both groups, we need approximately 323 patients to find differences in our primary outcome, effective communication, which involves open-ended questions, reflective statements, empathic responses to empathic opportunities, and eliciting questions. We assume that enrolled patients of the same physician will be correlated, and the level of intraphysician or intracluster correlation will be low to moderate. We assume a 20% difference for the primary outcome of the 4 communication behaviors (aim 1) between minorities (Black and Hispanic people) and nonminorities based on our pilot and the literature demonstrating similar differences in patient-centered communication for Black and Hispanic people [17]. Assumptions regarding psychological distress and satisfaction with communication (aim 2) and the distribution of pain outcomes (aim 3) have been drawn from our prior studies or the literature [20,32]. We assume that all patients should have their pain assessed. As stated in the literature, pain management (requiring opioids) and pain control will be applicable to 80% of enrolled patients. Assuming a range of intraphysician or intracluster correlations from 0.001 to 0.010, power ranges from 0.81 to 0.99 indicate sufficient power to detect the effect of implicit bias for Black versus White people and Hispanic versus Black people regarding patient-centered communication, pain outcomes, psychological distress, and satisfaction with communication.

## Results

This study received funding from the National Cancer Institute in March 2021. Patient and oncologist enrollment began in March 2022. We have recruited all 65 oncologists in the study, and patient recruitment is ongoing. Due to a lack of diversity among oncologists at both MSHS and DUHS, almost all enrolled oncologists identify as non-Hispanic White. There are 8 clinics located at DUHS and 8 at MSHS: gastrointestinal, sarcoma, melanoma, neuro-oncology, thoracic, breast, genitourinary, gynecologic, and head and neck. The study team plans to continue to enroll patients until March 2025. As of December 2024, we have enrolled 245 patients. We expect to publish the findings in October 2026.

## Discussion

### Principal Considerations

We anticipate that we will find that implicit bias among oncologists causes racial and ethnic differences in patient-centered communication and is associated with higher levels of psychological distress and lower levels of satisfaction with communication. We also anticipate that we will find that implicit bias among oncologists causes racial and ethnic gaps in pain assessment, which can lead to poor perception of pain control among minority patients with advanced solid cancer. Our findings will deepen our understanding of relationships among implicit bias, communication processes, management, and patient outcomes. This study is innovative because we assess implicit bias among oncologists who treat Hispanic patients and examine the goals of care over time using multiple postscan visits and audio recordings. No other team has assessed how the goals of care conversations evolve over multiple encounters. We outline the limitations and solutions below.

### Limitations

#### Limitation #1: Incomplete Number of Recordings Being Collected From Each Patient

We planned to obtain 3 audio recordings per patient to assess how the goals of care conversations change over time as the disease potentially progresses. However, we encountered difficulties in recording 3 encounters as some patients entered hospice or died.

#### Solution #1: Modifying Expectations for Analysis

We assessed the goals of care conversations at the first audio-recorded visit for all participants and realized that we would only be able to examine changes over time in a subset of patients. Given the prevalence of patients becoming too sick to continue participating, we are not replacing recordings.

#### Limitation #2: Difficulty Identifying Scans and Cancer Stage

Staff at the MSHS experienced challenges identifying eligible participants, specifically those with advanced cancer coming for a postscan visit, as the data were not readily available in the patient’s EHR. Unlike the DUHS, where the EHR includes stage information, at the MSHS, the EHR does not include this information, and thus, a labor-intensive manual review of all scheduled patients is needed. The manual review not only introduces a considerable workload for the staff but also poses a risk of oversight or delays in identifying eligible participants. The complexity of this task is compounded by the inherent variability in how medical information is recorded and cataloged within the EHR, making the identification of specific criteria, such as cancer stage, a labor-intensive and error-prone process.

#### Solution #2: Implementing Machine Learning–Based Approaches

To address this challenge, we are implementing a machine learning–based solution leveraging large language modeling. This approach offers several advantages over manual review. First, it eliminates the need for labor-intensive manual chart reviews, reducing the workload for staff and mitigating the risk of oversight or delays in identifying eligible participants. Additionally, by leveraging natural language processing techniques, the model can account for the inherent variability in how medical information is recorded and cataloged within the EHR, enhancing accuracy and efficiency in identifying specific criteria such as cancer stage. Furthermore, by integrating this solution into existing workflow systems, such as appointment scheduling software, we can automate the identification process, providing real-time eligibility screening for scheduled patients. This not only streamlines the recruitment process but also enables proactive identification of eligible participants, allowing for timely intervention and enrollment in the research protocol.

#### Limitation #3: Advanced Practice Providers and Fellows Have Postscan Conversations With Patients Instead of Oncologists

Oncologists at both sites had postscan conversations with advanced practice providers (nurse practitioners and physician assistants) and oncology fellows in practice. During instances where advanced practice providers and fellows see patients along with the consented oncologists, the oncologists might not say much in the encounter. Having multiple health care professionals in these encounters impedes the study’s objective of specifically recording conversations between patients and their oncologists who are enrolled in the study.

#### Solution #3: Making Requests to Oncologists, Fellows, and Advanced Practice Providers in Advance

When possible, the study coordinator emails oncologists in advance of the scheduled encounter. Often, oncologists agree to see the patients for the postscan conversation instead of their advanced practice providers or fellows. Third-party consents are obtained from advanced practice providers and fellows who may be a part of the recorded conversation.

#### Limitation #4: Differences in Racial or Ethnic Characteristics in the Patient Panels of Consented Oncologists

Our goal is to recruit 5-6 patients per oncologist. The DUHS currently has an equal number of Black and White patient participants recruited for the study owing to a low number of Hispanic patients who meet the eligibility criteria. Some oncologists see more Black patients than White patients, while others see more White patients than Black patients. At the MSHS, there is a higher number of patients who identify as Hispanic.

#### Solution #4: Meeting the Goals for Racial and Ethnic Diversity Across Sites

Due to the low number of eligible Hispanic patients at the DUHS, we are oversampling Hispanic patients at the MSHS. At the DUHS, the study team has been recruiting 3 Black patients and 3 White patients per oncologist, where possible. There have been some oncologists who see more Black patients and those who see fewer. We compensate for this by overrecruiting Black patients for some oncologists.

### Conclusion

This is one of the first studies to examine the goals of care over time in a diverse sample of patients with advanced solid tumors. A more in-depth understanding of the reasons underlying the disparities in care will be an important step toward improving the outcomes of over 65,000 minority patients diagnosed with advanced cancer each year. The knowledge generated by our project could inform both oncologist-level and patient-specific interventions.

This study aims to evaluate the role of oncologists’ implicit bias in racial and ethnic disparities in patient-centered communication, psychological distress, satisfaction with communication, pain assessment, and pain management among patients with advanced solid cancer. By collecting one of the largest datasets of conversations between oncologists and Black and Hispanic patients to date, this study lays the groundwork for developing interventions aimed at improving communication and care delivery. The significance of this study lies in its potential to deepen our understanding of the complex relationships among implicit bias, communication processes, management strategies, and patient outcomes. The findings generated by this project have the potential to inform both physician-level educational programs and patient-specific activation interventions, ultimately contributing to the reduction of disparities in cancer care.

In summary, this study has far-reaching implications for advancing equitable cancer care and underscores the importance of addressing implicit bias in health care delivery. By identifying and addressing barriers to effective communication and care, we can strive toward achieving better outcomes for all patients, regardless of race or ethnicity.

## Appendix

Multimedia Appendix 1 Clinician baseline survey.

Multimedia Appendix 2 Clinician postencounter survey.

Multimedia Appendix 3 Patient prescreener survey.

Multimedia Appendix 4 Patient baseline survey.

Multimedia Appendix 5 Patient postaudio survey.

## Abbreviations

DUHS: Duke University Health System
EHR: electronic health record
IAT: Implicit Association Test
IRB: institutional review board
MSHS: Mount Sinai Health System

## Data Availability

This study is being conducted in accordance with the following publication and data sharing policies and regulations:

National Institutes of Health (NIH) Public Access Policy, which ensures that the public has access to the published results of NIH-funded research. It requires scientists to submit final peer-reviewed journal manuscripts that arise from NIH funds to the digital archive PubMed Central upon acceptance for publication.
