# Supplementary figures and images for: Examining Potential Implicit Bias in Oncologist-Patient Communication (CONNECT): Protocol for an Observational 2-Site Study
Source: JMIR Res Protoc. 2025 Aug 14;14:e66086. doi: 10.2196/66086 (PMC12395106; doi:10.2196/66086)

Multimedia Appendix 1

**
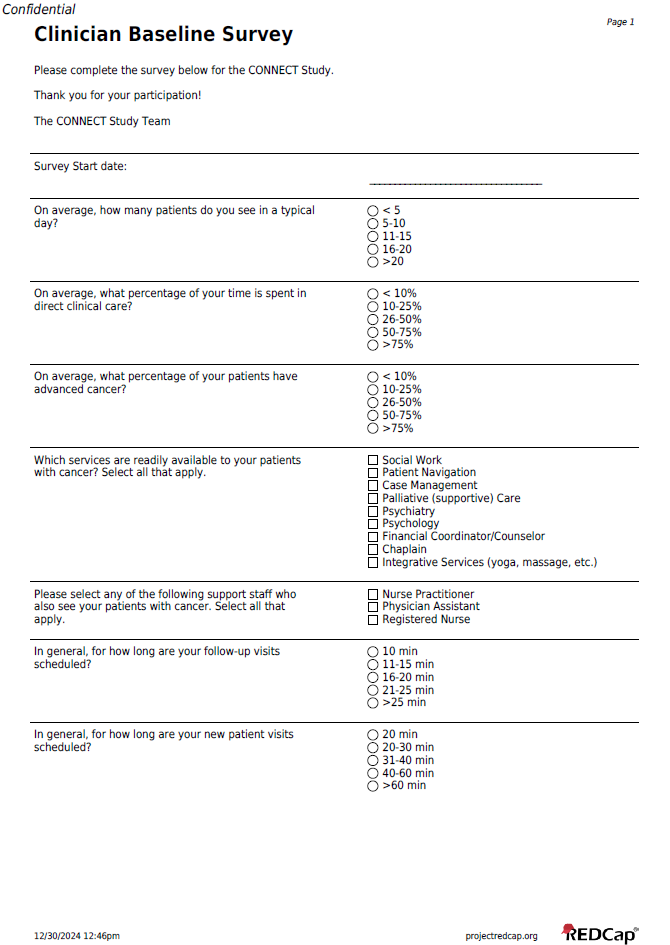
**

**
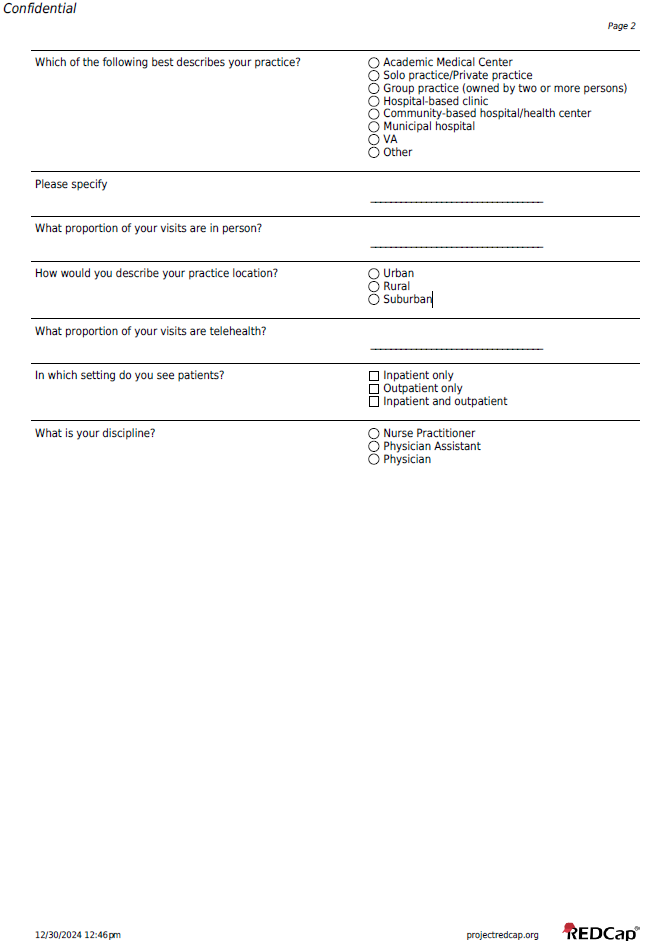
**

Supplement: Multimedia Appendix 1 [file resprot_v14i1e66086_app1.docx]

Multimedia Appendix 2

**
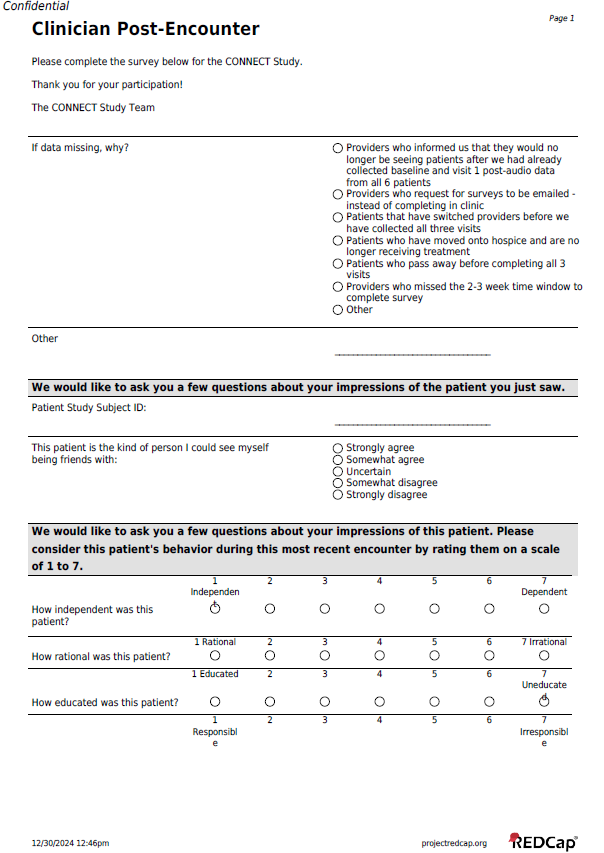
**

**
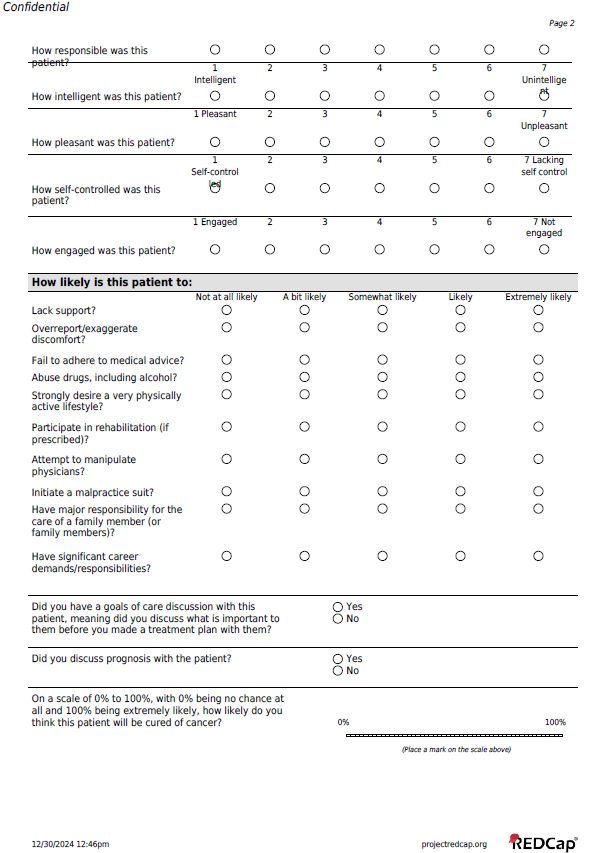
**

**
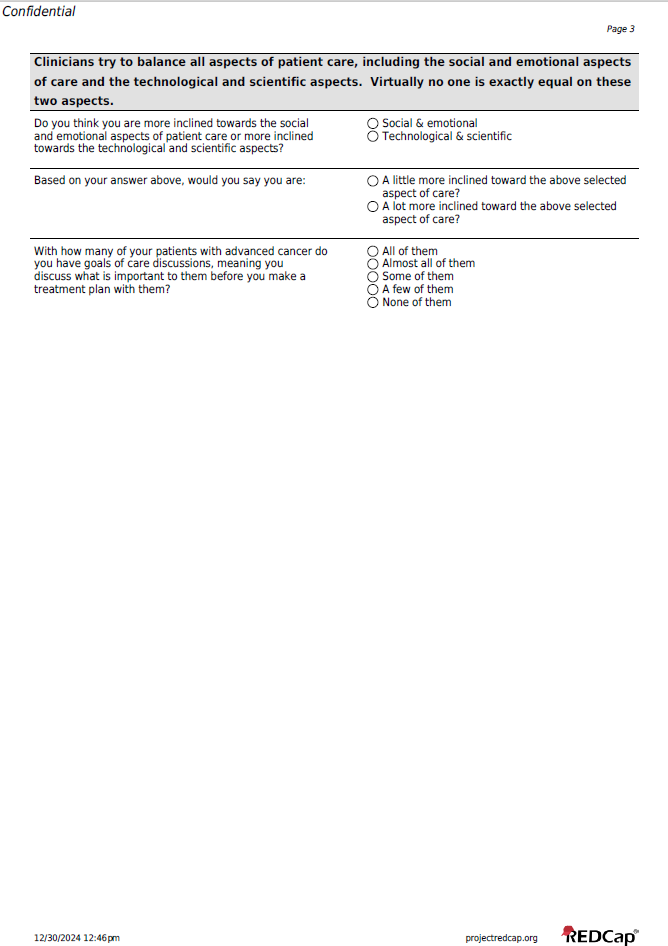
**

**
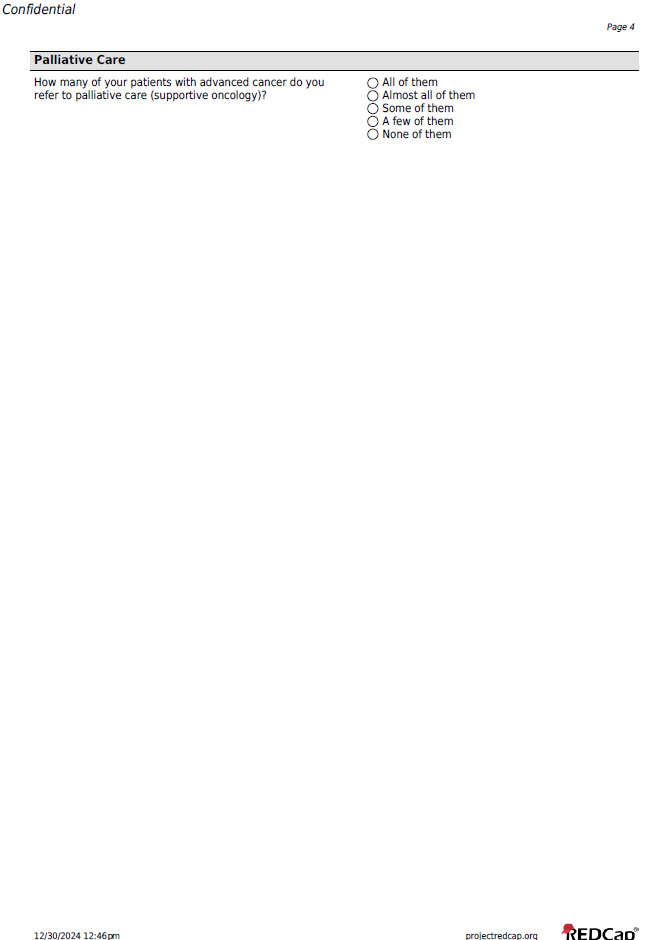
**

**
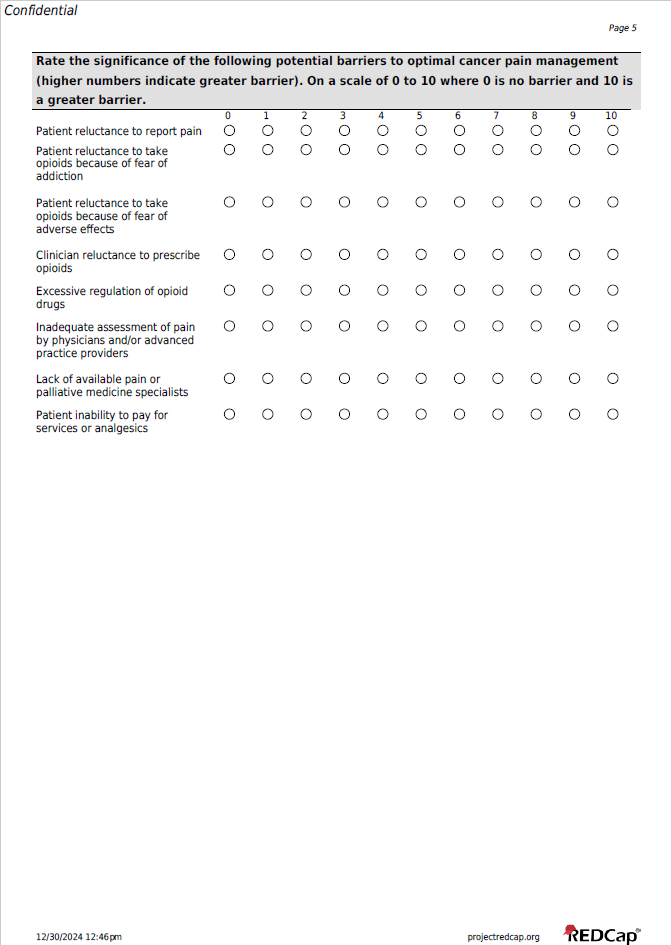
**

**
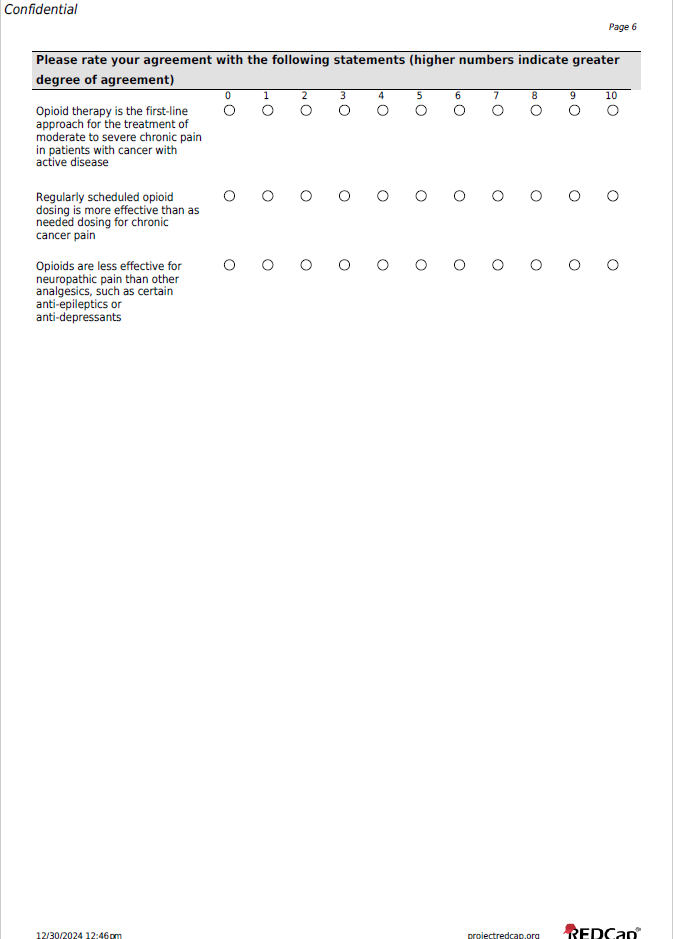
**

**
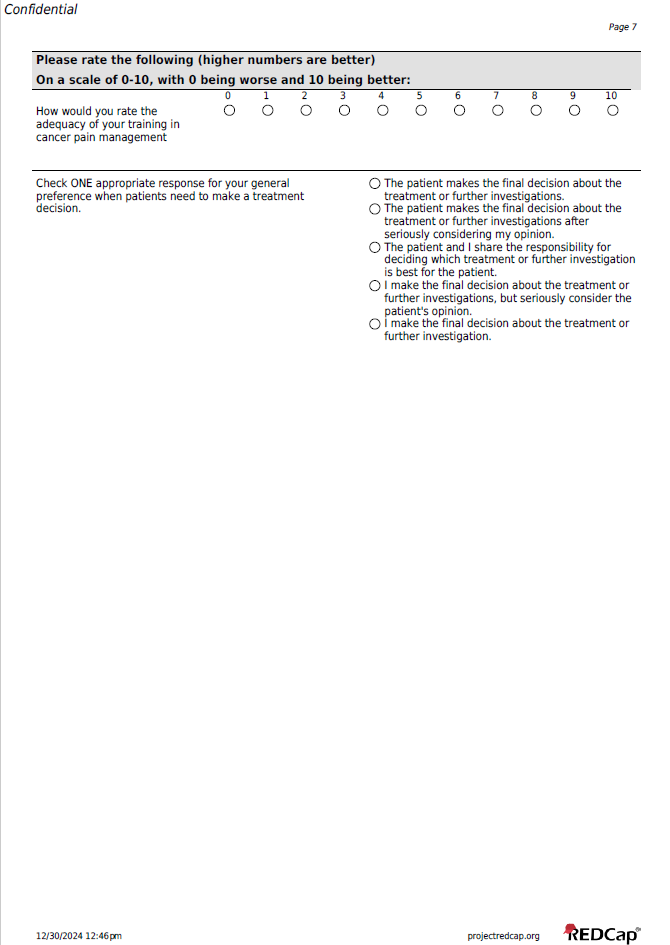
**

**
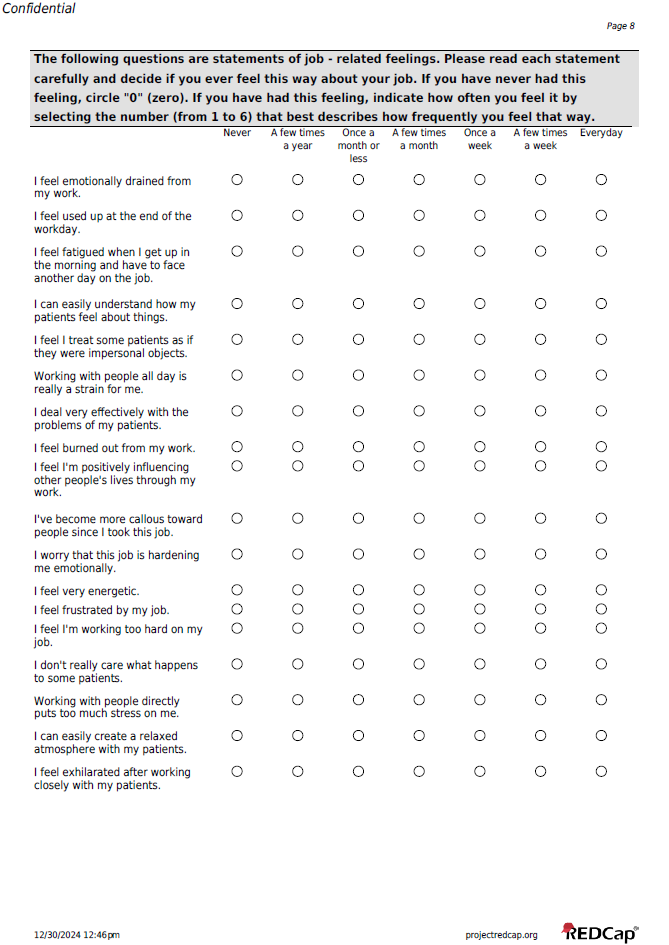
**

**
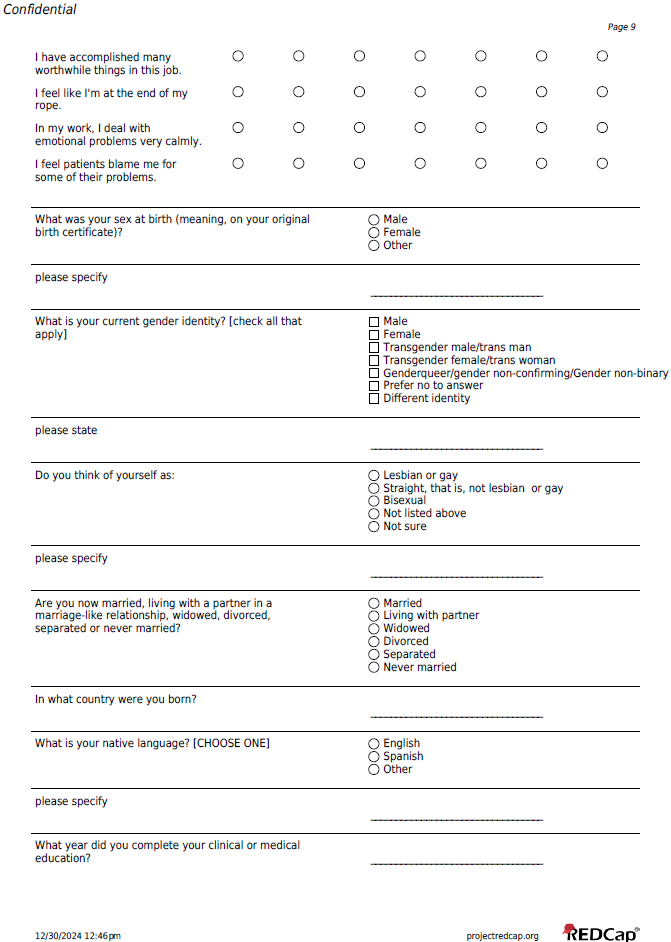
**

**
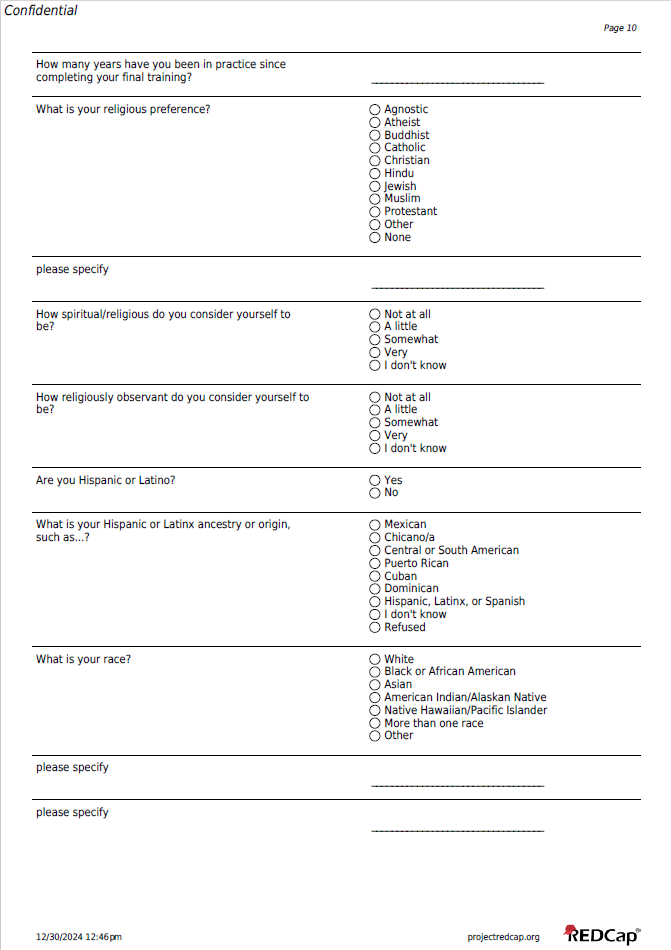
**

Supplement: Multimedia Appendix 2 [file resprot_v14i1e66086_app2.docx]

Multimedia Appendix 3


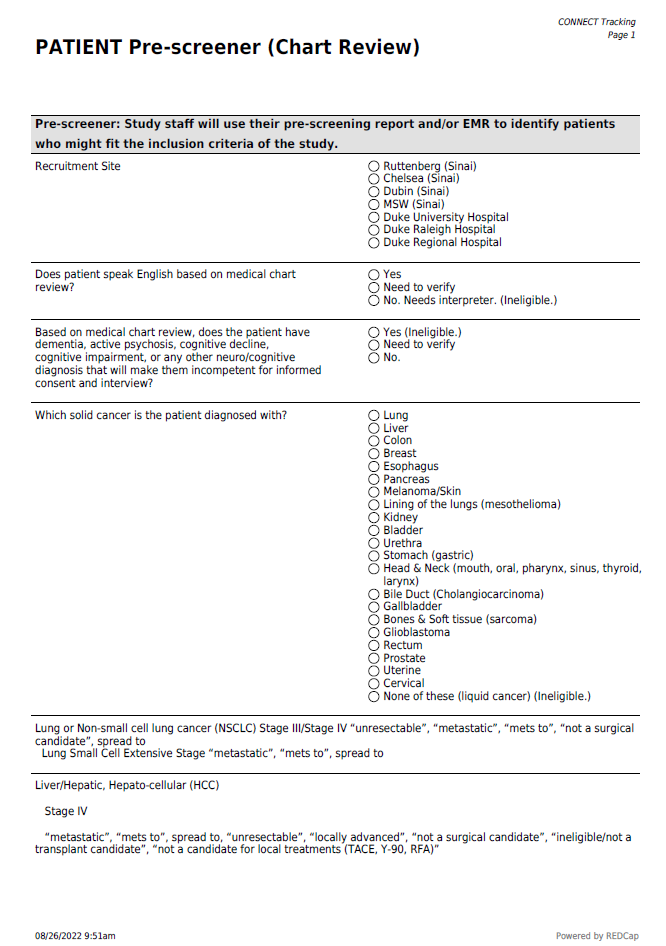


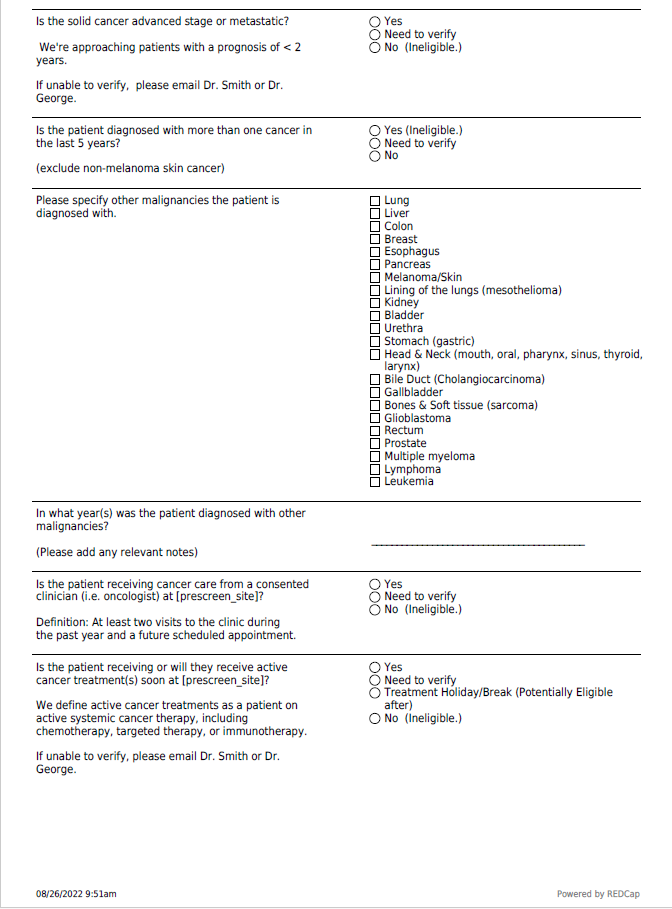


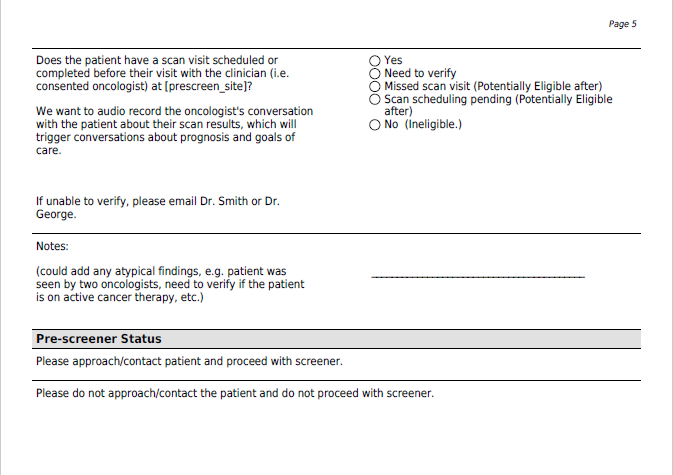

Supplement: Multimedia Appendix 3 [file resprot_v14i1e66086_app3.docx]

Multimedia Appendix 4


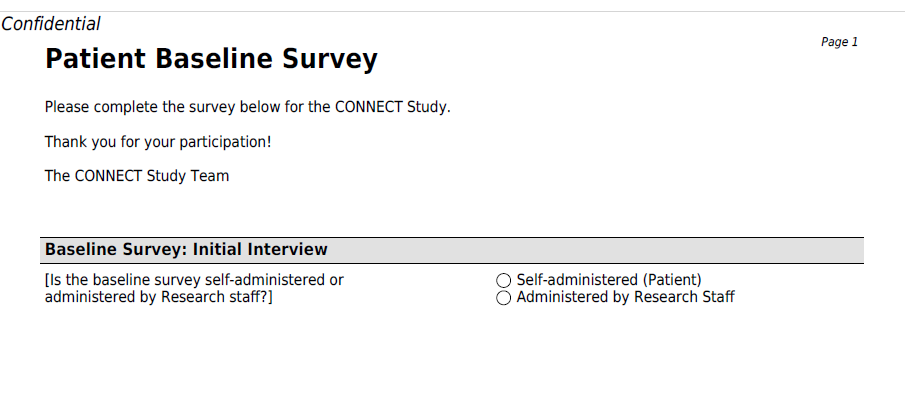


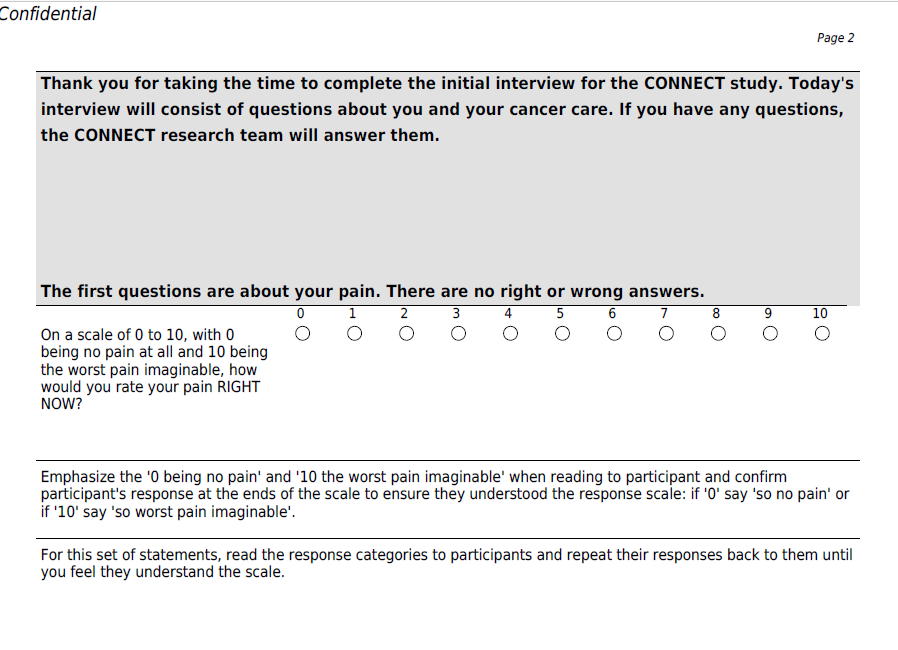


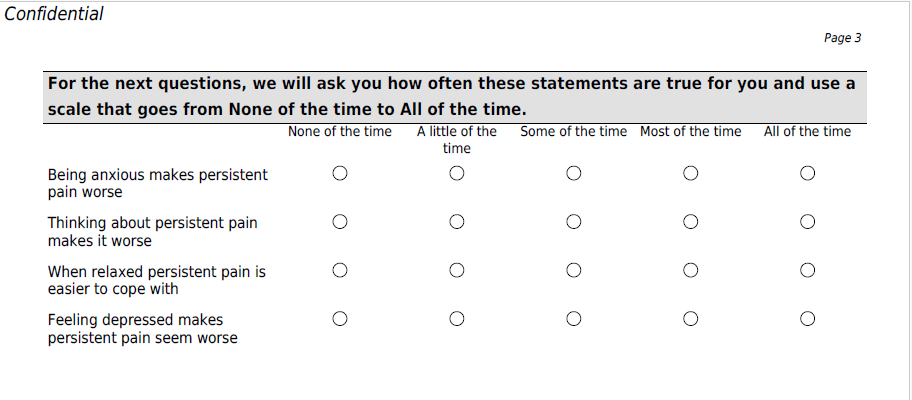


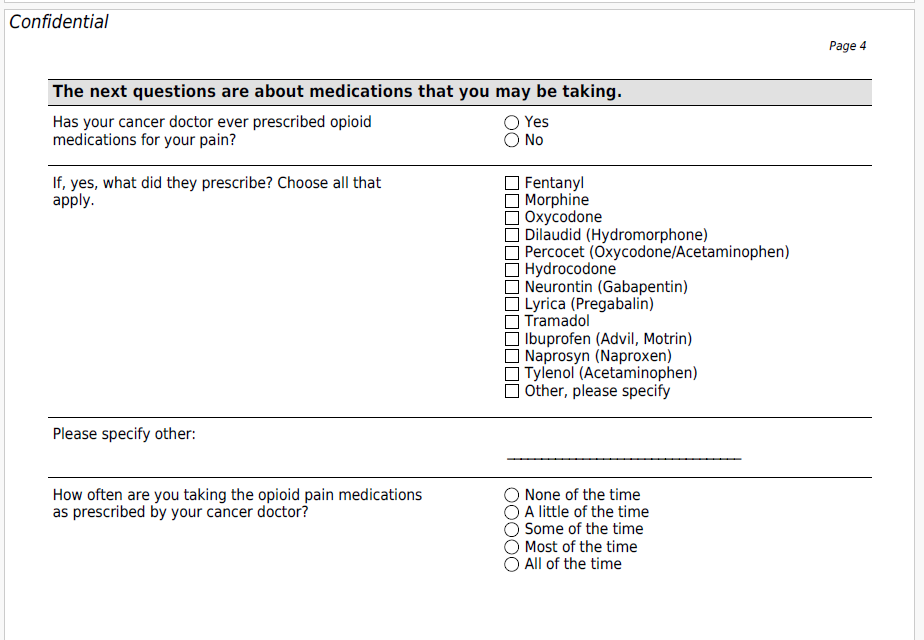


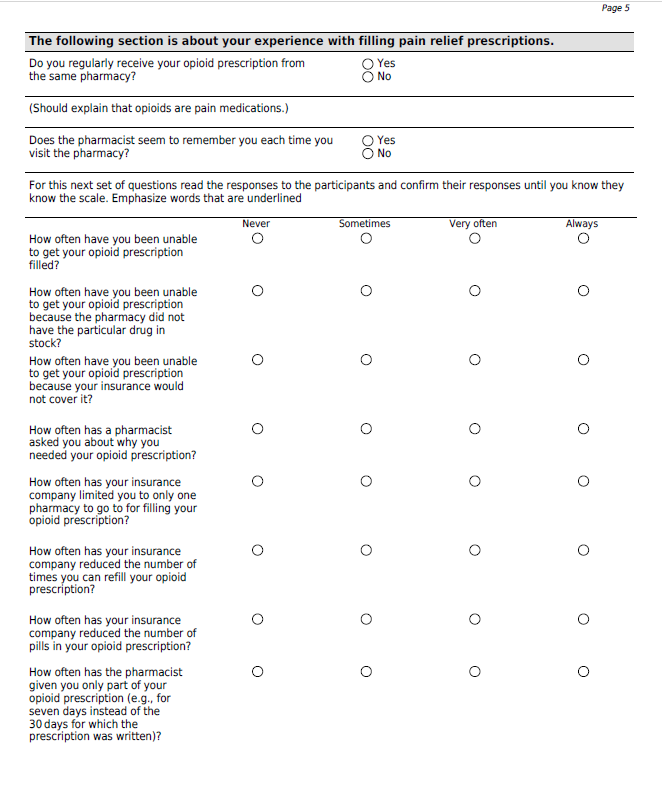


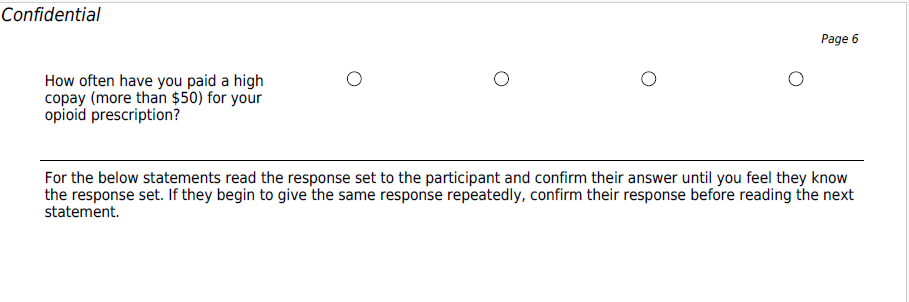


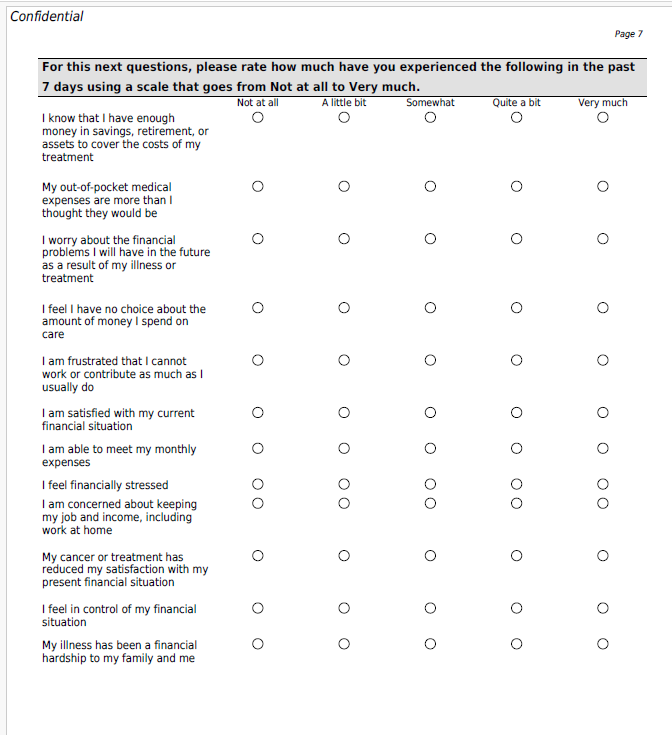


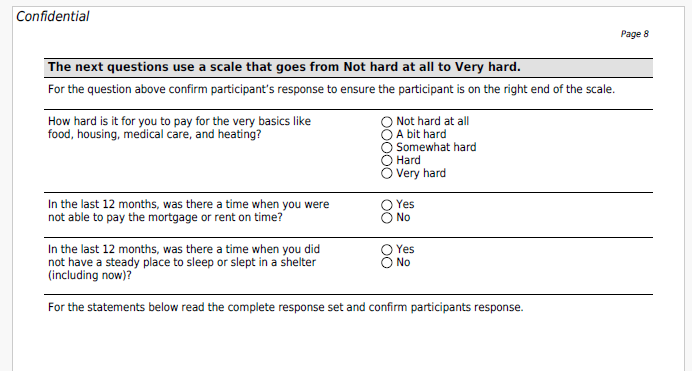


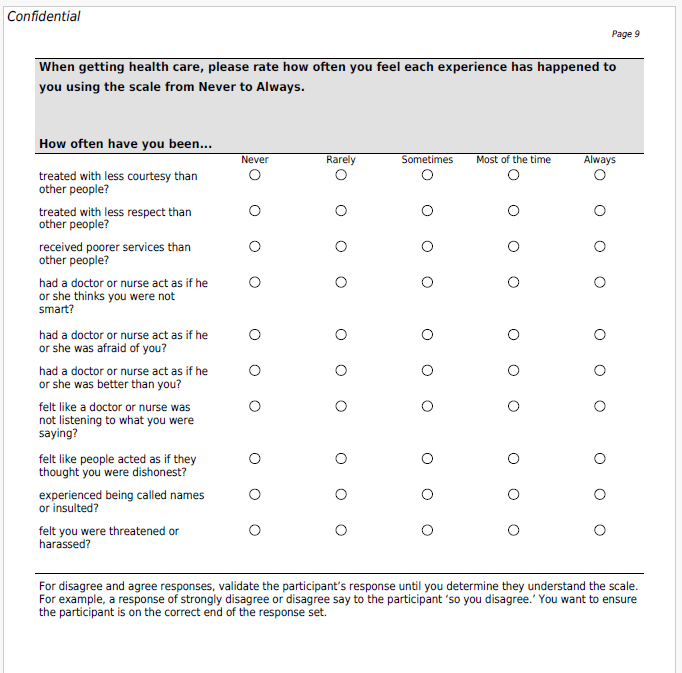


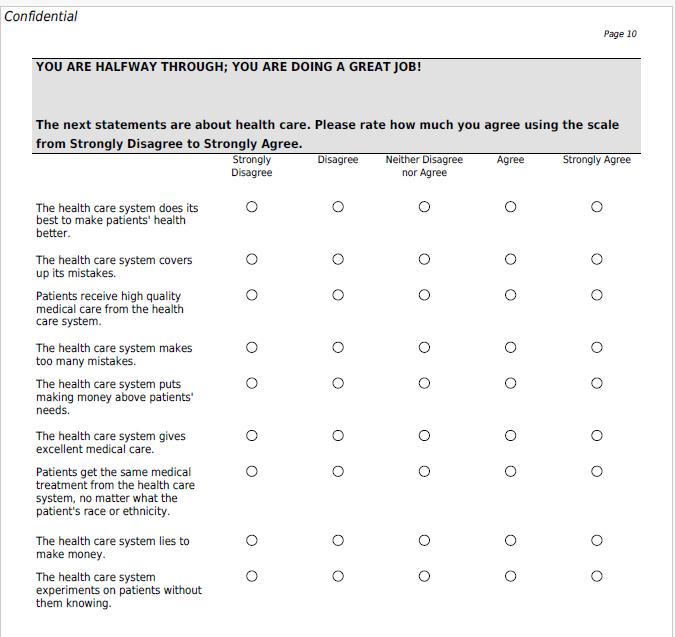


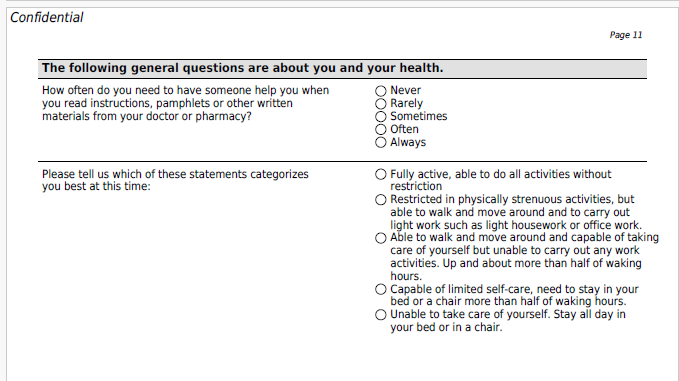


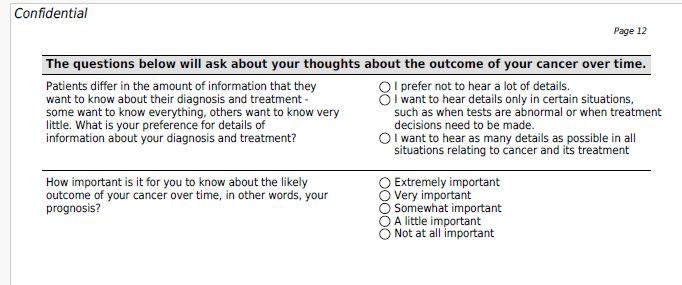


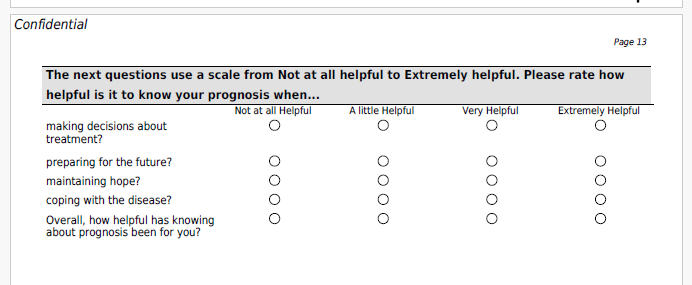


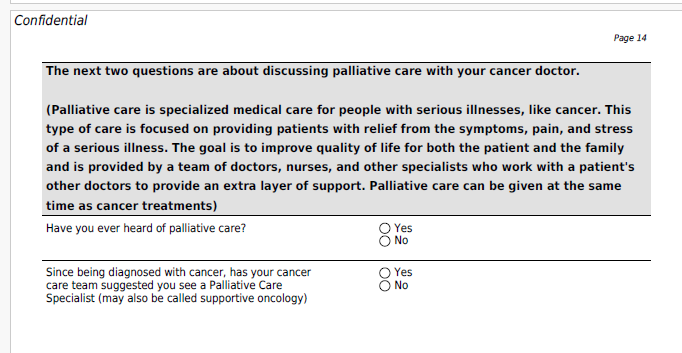


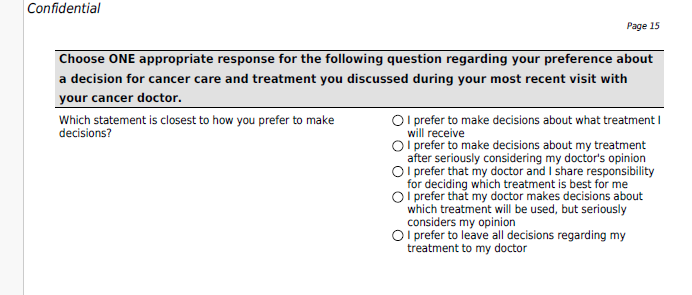


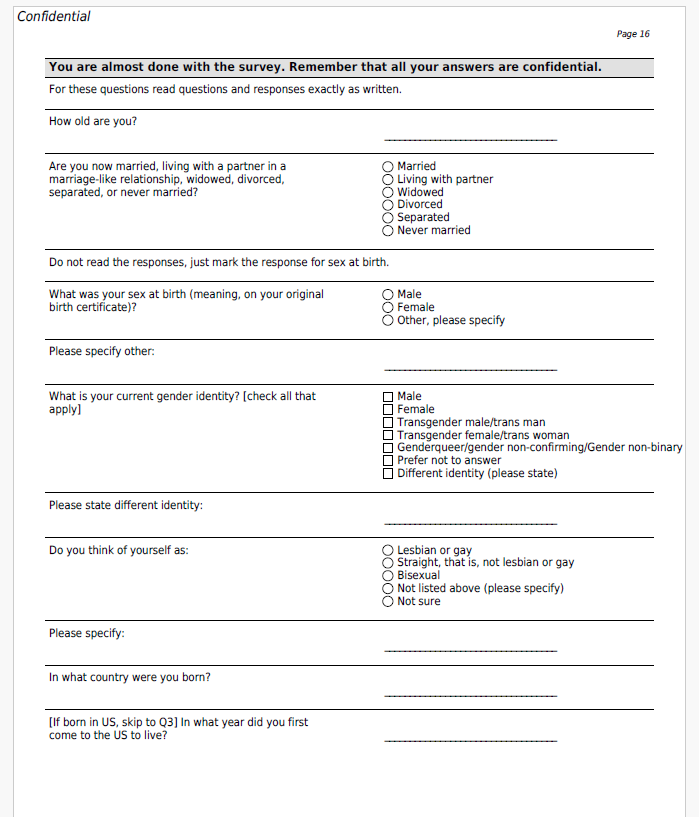


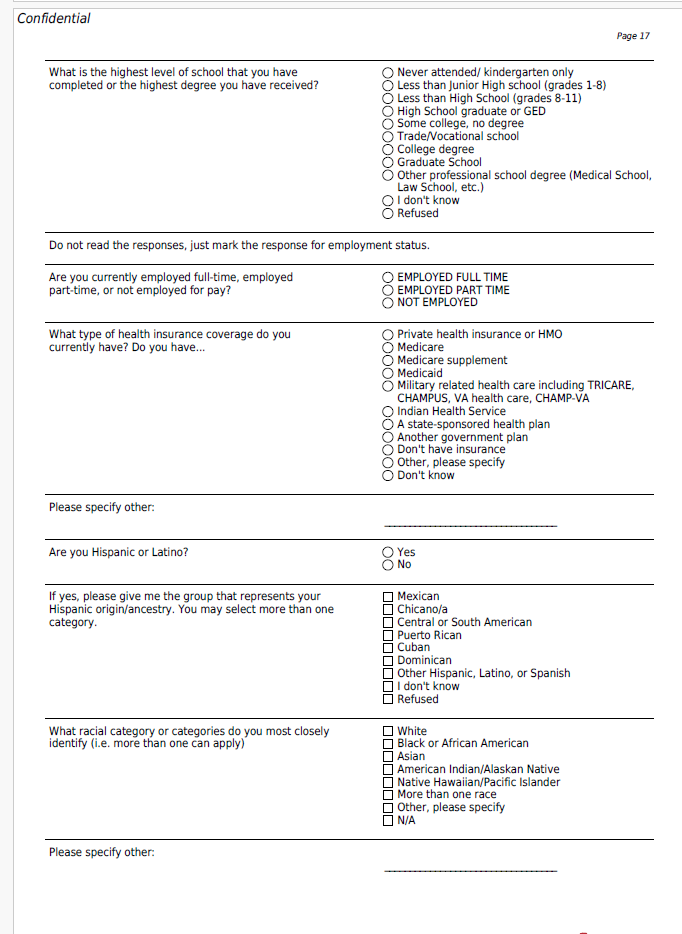


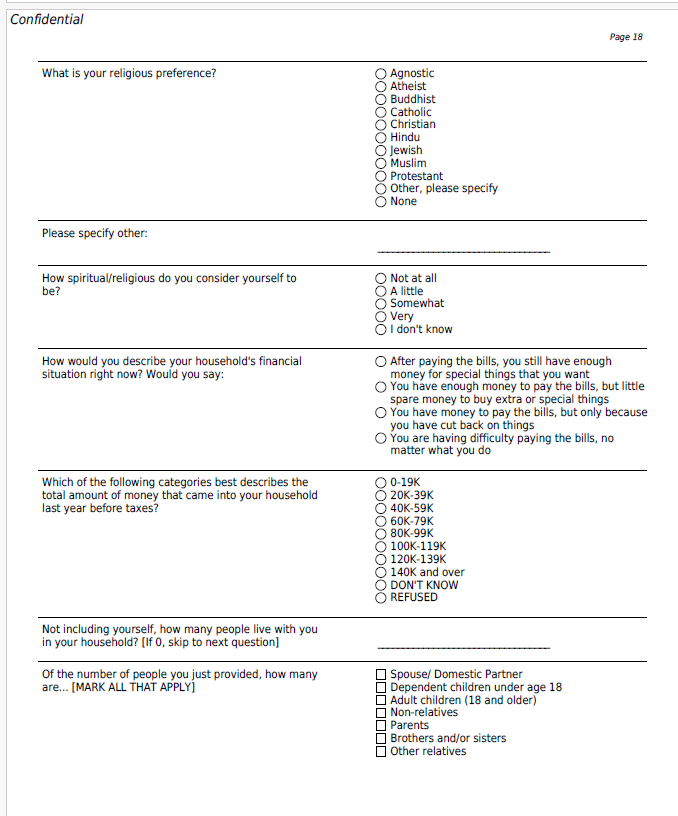


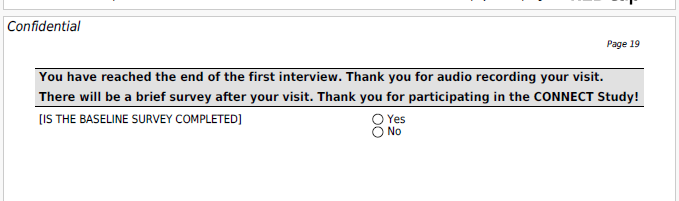

Supplement: Multimedia Appendix 4 [file resprot_v14i1e66086_app4.docx]

Multimedia Appendix 5

**
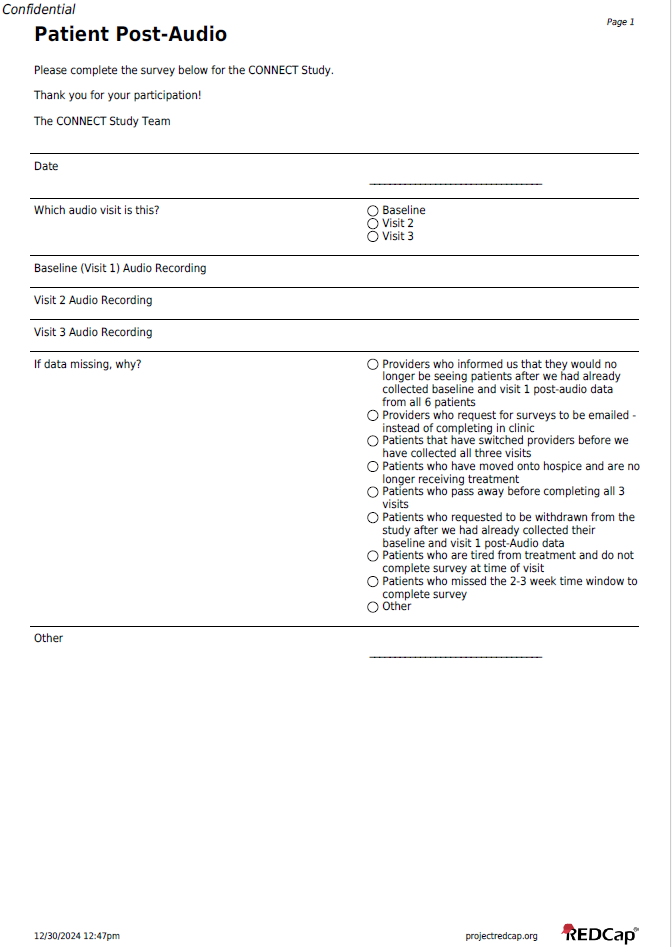
**

**
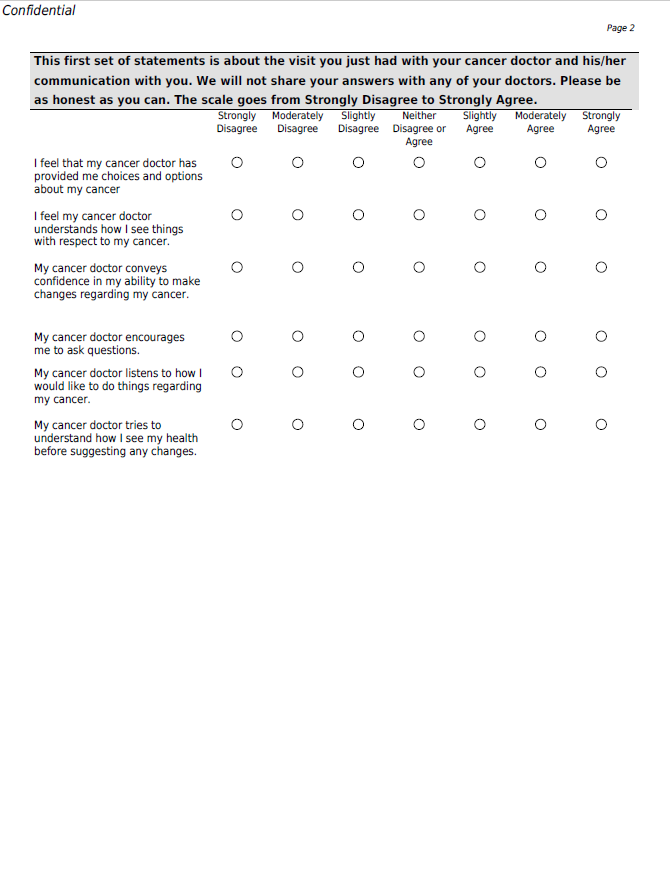
**

**
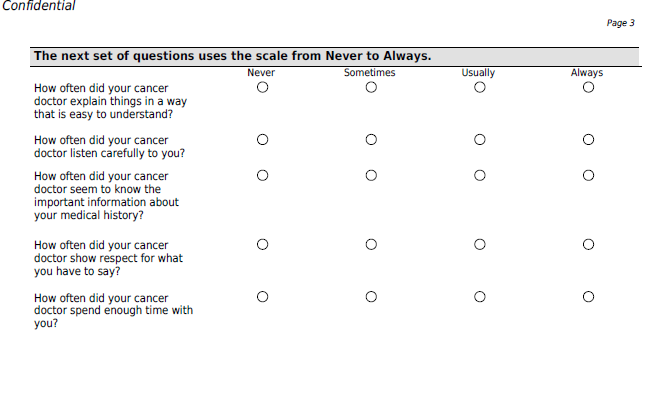
**

**
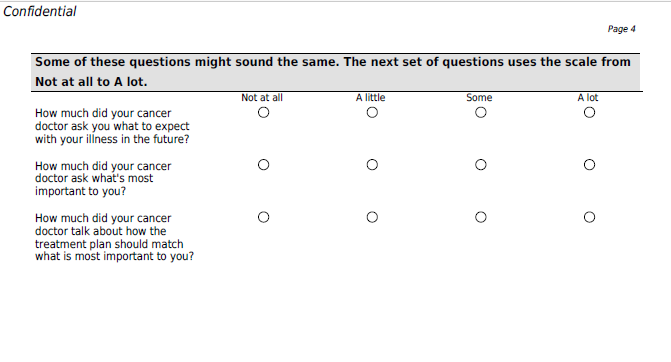
**

**
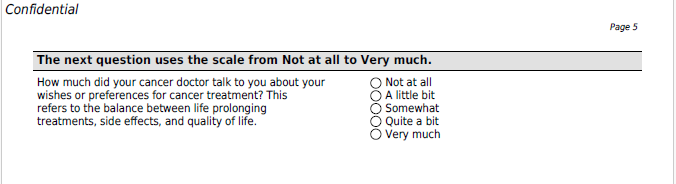
**

**
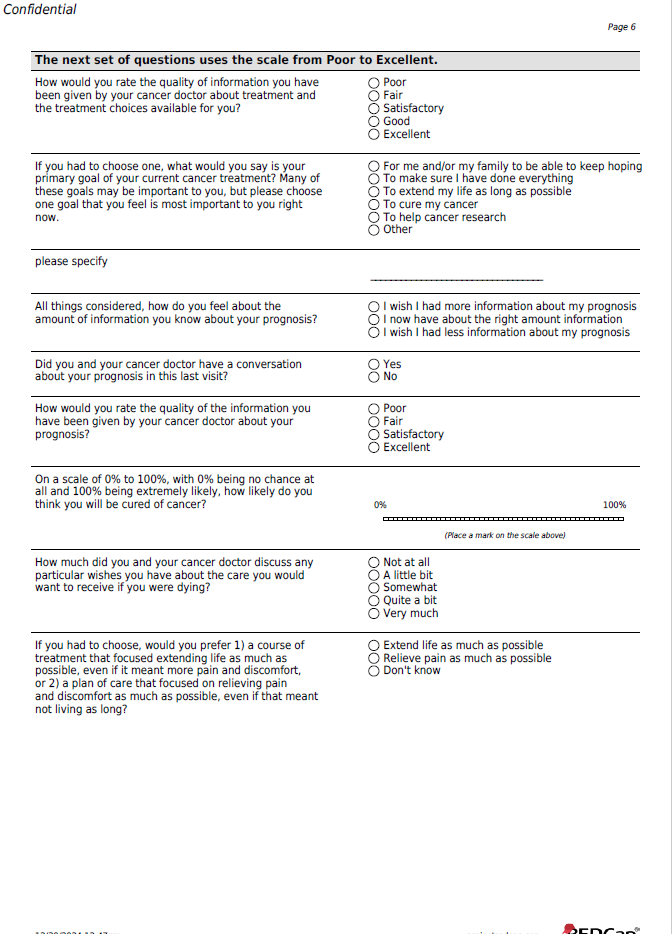
**


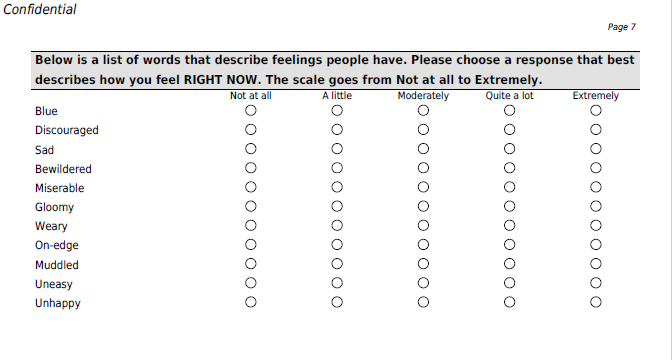


**
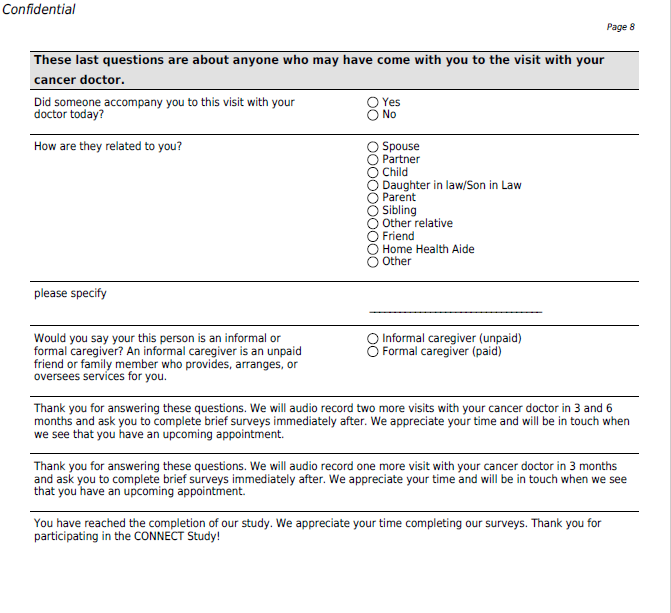
**

Supplement: Multimedia Appendix 5 [file resprot_v14i1e66086_app5.docx]
